# Lying-down nystagmus and head-bending nystagmus in horizontal semicircular canal benign paroxysmal positional vertigo: are they useful for lateralization?

**DOI:** 10.1186/1471-2415-14-136

**Published:** 2014-11-20

**Authors:** Jung-Hwan Oh, Sook-Keun Song, Jung Seok Lee, Jay Chol Choi, Sa-Yoon Kang, Ji-Hoon Kang

**Affiliations:** Department of Neurology, Jeju National University Hospital, Ara 1-dong, Jeju-si, Jeju, 690-767 South Korea

**Keywords:** Benign paroxysmal positional vertigo, Nystagmus, Semicircular canals

## Abstract

**Background:**

Lateralization of horizontal semicircular canal benign paroxysmal positional vertigo (HSC-BPPV) is very important for successful repositioning. The directions of lying-down nystagmus (LDN) and head-bending nystagmus (HBN) have been used as ancillary findings to identify the affected sites. This retrospective study was performed to evaluate the lateralizing values of LDN and HBN using clinical and laboratory findings for lateralizing probabilities in patients with HSC-BPPV.

**Methods:**

For 50 HSC-BPPV patients with asymmetric direction-changing horizontal nystagmus (DCHN) during the head-rolling test (HRT) using Frenzel goggles, the directions of LDN and HBN were evaluated and compared to those determined by video-oculography. Directional LDN was defined as the contralesional direction of nystagmus in geotropic types and the ipsilesional direction in apogeotropic types. Directional HBN was defined as the opposite direction relative to directional LDN. We also analyzed LDN and HBN in 14 patients with a history of ipsilesional peripheral vestibulopathy, caloric abnormality or conversion from other types of BPPV (such as probable localized HSC-BPPV, pro-BPPV).

**Results:**

LDN and HBN were seen in 68% (34/50) and 76% (38/50) of patients, respectively. Of these, 19 (55.9%), and 28 (73.7%) patients showed directional LDN and HBN, respectively. The proportion of patients with directional LDN and HBN was much smaller among the pro-BPPV patients (4/12 for LDN, 3/10 for HBN).

**Conclusions:**

LDN and HBN did not seem to predict lateralization in patients with HSC-BPPV. To improve the prediction of lateralization of HSC-BPPV, it is necessary to modify the maneuvers used to elicit LDN or HBN, especially in cases of symmetric DCHN during HRT.

## Background

**Figure 1:**
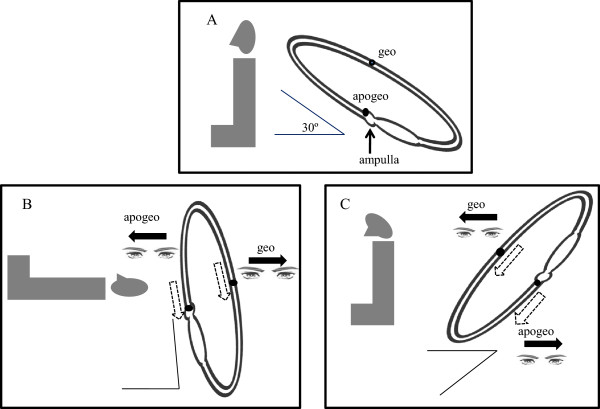
**Lying-down nystagmus and head-bending nystagmus in right horizontal semicircular canal benign paroxysmal positional vertigo.** The horizontal semicircular canal is at an ~30 angle from the horizontal plane in the normal sitting position **(A)**. Ampullopetal endolymphatic flow induces the ipsilesional direction of lying-down nystagmus(LDN) in apogeotropic horizontal semicircular canal benign paroxysmal positional vertigo (HSC-BPPV), whereas LDN beats toward the contralesional side are caused by the ampullofugal direction of endolymphatics in the geotropic type **(B)**. The directions of head-bending nystagmus (HBN) and LDN are opposite to each other **(C)**. (Adapted from reference [23]: G. Asprella-Libonati. Pseudo-Spontaneous Nystagmus: a new sign to diagnose the affected side in Lateral Semicircular Canal Benign Paroxysmal Positional Vertigo. Acta otorhinolaryngologica Italica : organo ufficiale della Societa italiana di otorinolaringologia e chirurgia cervico-facciale, Figure one, three 2008, 28(2):73–78., with permission, Copyright © by Società Italiana di Otorinolaringologia e Chirurgia Cervico-Facciale Via Luigi Pigorini, 6/3 00162 Roma, Italy).

Benign paroxysmal positional vertigo (BPPV) is the most common cause of peripheral vertigo, and is characterized by brief recurrent episodes of vertigo triggered by changes in position [1, 2]. Posterior semicircular canal benign paroxysmal positional vertigo (PSC-BPPV) is the most common BPPV condition [2]. The incidence of horizontal semicircular canal BPPV (HSC-BPPV) is reported to range between 10 and 42.7% as the BPPV definition has been newly established and nystagmus recording techniques have advanced in recent years [3]. HSC-BPPV is characterized by a direction-changing horizontal nystagmus (DCHN) during turning the head by 90° to either side in the supine position (head-rolling test, HRT) [1, 2]. The nystagmus can be either always toward the ground (“geotropic”) or the ceiling (“apogeotropic”) [4]. Geotropic nystagmus is caused by free-floating otolithic debris accumulating in the endolymph of the horizontal semicircular canal (canalolithiasis) [5–8]. Apogeotropic nystagmus is likely caused by detached debris that adheres to the cupula (cupulolithiasis) [5, 7, 9]. According to Ewald’s second law, “ampullopetal endolymphatic flow produces a stronger response than ampullofugal flow in the horizontal semicircular canal”, evoked nystagmus is stronger when the head is turned toward the affected side in geotropic HSC-BPPV. On the contrary, turning the head toward the healthy side induces a stronger nystagmus in apogeotropic HSC-BPPV [10–12]. Canalith repositioning maneuvers, such as Lempert maneuver, barbecue rotation, forced prolonged position, Vannucchi–Asprella maneuver, and Gufoni maneuver are effective in geotropic HSC-BPPV [13–16]. Head-shaking maneuver is effective in the apogeotropic type [3, 14, 17], but other maneuvers such as the modified Semont maneuver [17, 18] or Gufoni maneuver [19, 20] can also be used. Lateralization of lesions is critical for effective treatment. However, lateralization is often difficult due to similar DCHN intensity during HRT in clinical practice. According to recent studies, the directions of nystagmus induced by fast lying down from the sitting position (lying-down nystagmus, LDN) and rapid head bending toward the pitch axis (head-bending nystagmus, HBN) aid the lateralization of HSC-BPPV [4, 21, 22]. The authors of these studies suggested that head-bending nystagmus was mostly directed toward the affected side in geotropic HSC-BPPV while toward the healthy side in apogeotropic type, and the directions of HBN and LDN were opposite (Figure 1). However, those patterns of nystagmus are not always detected in the actual clinical setting, and nystagmus can be directed toward the wrong directions for the lesions.

BPPV often recurs [15] and can be secondary to Meniere’s disease or vestibular neuritis [24]. Furthermore, PSC-BPPV can often be transformed to HSC-BPPV [15, 16], and canal paresis generated by impaired endolymphatic flow is reported to recover after cure of HSC-BPPV [16, 25–27]. Taking the clinical characteristics of BPPV into consideration, we conducted a retrospective review of clinical records and caloric tests of HSC-BPPV patients to assess HBN and LDN in lesion lateralization using asymmetric DHCN during HRT.

## Methods

### Subjects

**Figure 2:**
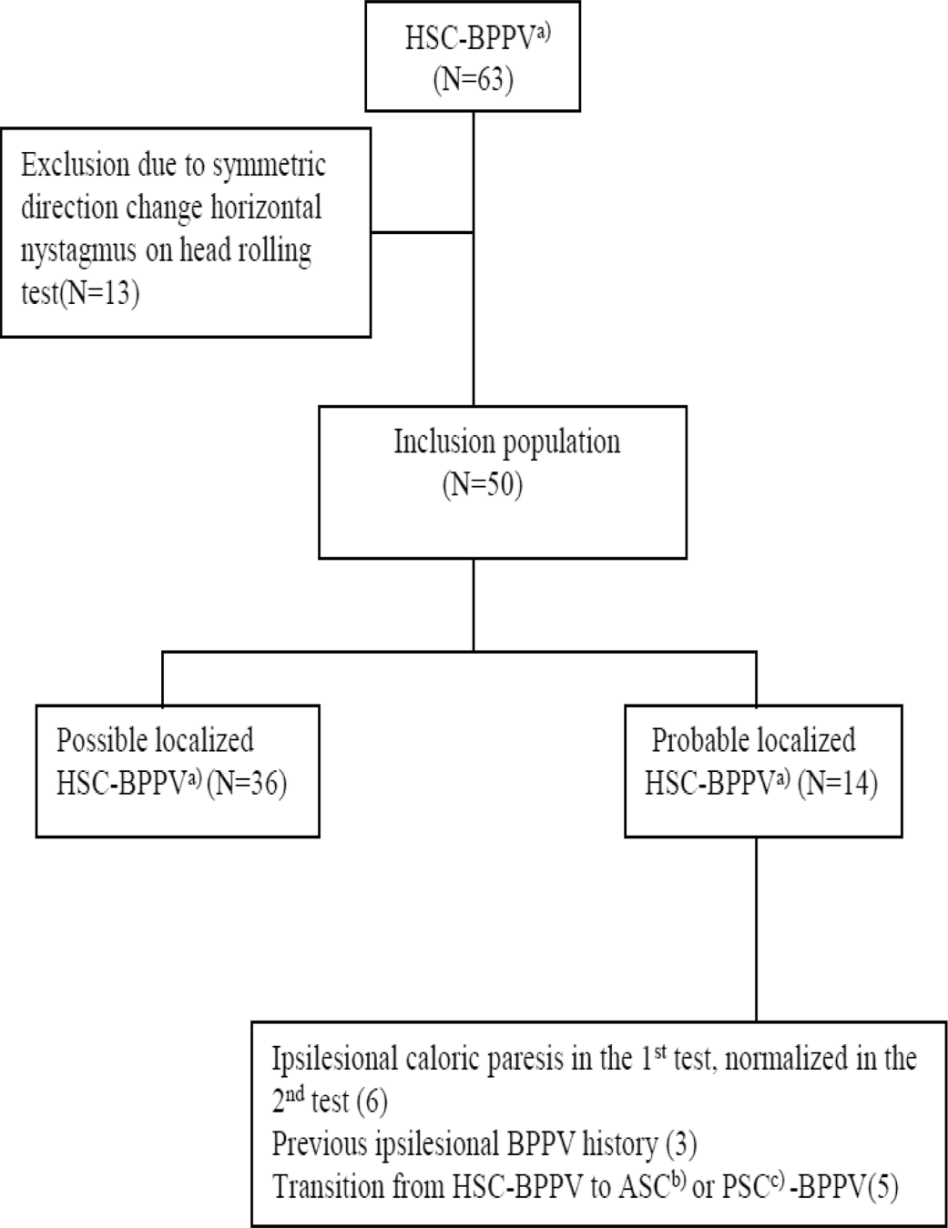
**Flow diagram of study population.** ^a)^Horizontal semicircular canal benign paroxysmal positional vertigo. ^b)^Anterior semicircular canal benign paroxysmal positional vertigo. ^c)^Posterior semicircular canal benign paroxysmal positional vertigo.

This study was performed after gaining institutional review board (IRB) approval of Jeju National University Hospital (JNUH) and involved patients diagnosed with HSC-BPPV according to electronic medical records (EMR) at Department of Neurology, Neuroscience Center at JNUH from February 2010 to September 2011. A waiver of informed consent was also permitted by the IRB, owing to the retrospective nature of the study. The inclusion criteria were 1) a history of positional vertigo, 2) geotropic or apogeotropic nystagmus during the HRT, and 3) vertigo or nystagmus not caused by diseases of the central nervous system [28]. Among a total of 63 patients who met the inclusion criteria, 50 patients showed asymmetric direction changes in horizontal nystagmus during HRT. Thirteen patients showing no differences in DCHN during HRT were excluded (Figure 2).

### Positioning nystagmus test

Nystagmus was evaluated with infrared video-Frenzel goggles (Easy-Eyes®, SLmed, Seoul, Korea) in accordance with the practice guidelines for vertigo at Department of Neurology, JNUH. The HRT was performed in the supine position with the head elevated 30° from the horizontal. The head was turned by 90° to the left side for 50 s to examine the nystagmus. Then, the head was returned to the neutral position for 30 s. If the nystagmus was still observed in the neutral position, we waited until it disappeared. Then, the head was turned by 90° to the right side for 50 s and the nystagmus was examined. Subsequently, patients were asked to sit in the head-upright position with the eyes looking forward during 30 s and then to bend the head at 60° forward the pitch axis for 50 s for the observation of nystagmus (HBN). Thereafter, the patients were asked to take an upright-sitting position with the eyes looking forward for 30 s and then to quickly lie down for 50 s; the nystagmus (LDN) was then examined.

To discriminate between PSC-BPPV and anterior semicircular canal BPPV (ASC-BPPV), the Dix-Hallpike maneuver [29] was also performed on each side consecutively. Based on Ewald’s second law, lateralization of the lesion side was determined as the result of DCHN during HRT [10–12]. Directional HBN was defined as the nystagmus direction toward the affected ear in HSC-BPPV for geotropic types. Directional LDN was defined as the contralesional direction of nystagmus for the geotropic type. For apogeotropic types, directional HBN and LDN were in the opposite directions relative to the geotropic types [4, 21, 22].

### Recordings of nystagmus

Positioning nystagmus was also recorded using video nystagmography (SLVNG®, SLmed, Seoul, Korea). The exam procedure and definition of lateralization were the same as above. LDN and HBN were analyzed and the asymmetry of LDN or HBN is indicated by the symmetry index (SI) greater than 10%; SI is defined as follows:


where SPV is the slow phase velocity.

### Bithermal caloric test

An alternating bithermal caloric test was conducted with Air-star® (Micromedical Technologies, Belgium); the test was performed again when vertigo was completely cured. Nystagmus SPV was measured every 180 s in the following order: cold left, cold right, warm left, and warm right ears. The temperature of cold and warm air waves was 27°C and 48°C, respectively. Stimulation time was 50 s. The percentage of canal paresis was calculated using the Jonkee’s formula [26]; canal paresis was defined as a greater than 25% difference between in maximal nystagmus slow phase eye velocity between the right and left sides. Bilateral canal paresis was diagnosed when the sum of slow phase velocities for each ear was below 12°/s.

### Medical history

Medical history of BPPV, vestibular neuritis, Meniere’s disease, and peripheral vertigo was confirmed based on EMR.

### Selection of patients with high probability of lateralization

To thoroughly evaluate HSC-BPPV lateralization, the following criteria are suggested. Among 50 patients who met the inclusion criteria, probable localized HSC-BPPV(pro-BPPV) was diagnosed if at least one of the following conditions was satisfied, whereas possible localized HSC-BPPV(pos-BPPV) was diagnosed if none of them was met: 1) conversion into PSC- or ASC-BPPV on the lesion side after HSC-BPPV diagnosis, 2) canal paresis on the same side in the initial caloric test, and normal condition in the second test, 3) history of vestibular neuritis or Meniere’s disease according to EMR, or recurrence of PSC-, ASC- or HSC-BPPV on the same side.

### Statistical analysis

Descriptive statistics were presented for continuous variables according to each demographic variable, and frequency and percentage were presented for categorical variables. Cohen’s kappa coefficient was used to identify concordance for the direction of LDN and HBN between video-oculography and Frenzel goggles. Statistical analyses were conducted using SPSS version 18.0. Differences were considered statistically significant at *p* < 0.05.

## Results

### Demographic characteristics

Among 50 HSC-BPPV patients with asymmetric DHCN during HRT, 31 patients (62%) had geotropic types and 19 patients (38%) had apogeotropic types (Table 1). The study included 32 females (64%) and 18 males (36%) with mean age of 63.3 ± 14.1 years (range, 26–90). The patients visited our hospital within 2 h – 1 year of the onset of symptoms (median, 2 days). Most cases were idiopathic BPPV; in 2 patients, BPPV occurred immediately after head trauma. None of the patients had a history of vestibular neuritis or Meniere’s disease. Three patients had a history of PSC-BPPV on the ipsilesional side, and 5 patients had a conversion of HSC-BPPV into PSC-BPPV in the affected ear after diagnosis (Figure 2).

**Table 1:** **Direction of lying down nystagmus(LDN) and head bending nystagmus(HBN) in 50 patients**

		Geotropic (N = 31)	Apogeotropic (N = 19)	Total (N = 50)
LDN	Directional LDN	63.2%(12/19)	46.7%(7/15)	55.9%(19/34)
	Absent	38.7%(12)	21.1%(4)	32.0%(16)
HBN	Directional HBN	73.9%(17/23)	73.3%(11/15)	73.7%(28/38)
	Absent	25.8%(8)	21.1%(4)	24.0%(12)

### Bithermal caloric test

Bithermal caloric tests were performed in all patients and were repeated in all but one patient. The time interval between the first and second caloric tests ranged from 2 to 42 days (mean, 15.3; median, 12). None of the patients had bilateral canal paresis. Canal paresis was detected on the affected side in 13 patients in both caloric tests and in 6 patients only in the first test. In these 6 patients, canal paresis was normalized in the second test (Figure 2).

### Analysis of LDN and HBN

Among 50 patients, LDN was seen in 34 patients (geotropic, 19; apogeotropic, 15) and HBN was seen in 38 patients (geotropic, 23; apogeotropic, 15) (Table 1). Among 31 patients with lateralization to asymmetric geotropic DHCN during HRT, LDN was in the contralesional direction (directional) in 12 patients and in the ipsilesional direction (opposite directional) in 7 patients. No LDN was seen in 12 patients. Among 19 patients with lateralization to asymmetric apogeotropic DHCN during HRT, LDN was seen in 15 patients. LDN was ipsilesional (directional) in 7 patients and contralesional (opposite directional) in 8 patients. Of 31 patients with asymmetric geotropic DCHN, HBN was found in 23 (73.2%). HBN was ipsilesional (directional) in 17 (73.9%) and contralesional (opposite directional) in 6(26.1%) patients. Of 19 patients with asymmetric apogeotropic DCHN, HBN was seen in 15 patients; of these, contralesional (directional) nystagmus was found in 11 patients (73.3%) (Table 1). Pro-BPPV was found in 14 of 50 patients. LDN and HBN were seen in 85.7% (12/14) and 71.4% (10/14) of patients, and directional LDN and HBN were seen in 33.3% (4/12) and 30% (3/10) of patients, respectively (Table 2).

**Table 2:** **Direction of lying down nystagmus(LDN) and head bending nystagmus(HBN) in 14 patients with probable localized HSC-BPPV**

		Geotropic (N = 8)	Apogeotropic (N = 6)	Total (N = 14)
LDN	Directional LDN	33.3%(2/6)	33.3%(2/6)	33.3%(4/12)
	Absent	25.0%(2)	0.0%(0)	14.3%(2)
HBN	Directional HBN	20%(1/5)	40%(2/5)	30%(3/10)
	Absent	37.5%(3)	16.7%(1)	28.6%(4)

Video-oculography was performed in 40 patients. Ten patients who were not examined by video-oculography all had pos-BPPV. There was a highly significant coefficient of concordance between the directions of LDN and HBN measured with video-oculography and Frenzel goggles in all patients (*N* = 40, LDN, κ = 0.66; *p* = 0.00, HBN, κ = 0.84; *p* = 0.00), pro-BPPV patients (*N* = 14, LDN, κ = 0.67; *p* = 0.00, HBN, κ = 0.69; *p* = 0.01), and pos-BPPV patients (*N* = 26, LDN, κ = 0.65; *p* = 0.00, HBN, κ = 0.88; *p* = 0.00).

## Discussion

Lateralization of HSC-BPPV is determined based on the difference in DCHN intensity during HRT, and is critical in the choice of the therapeutic maneuver. However, it is often difficult to identify the difference in clinical practice. A number of studies have attempted to investigate lateralization of the lesion side on the basis of the various types of nystagmus manifested in different positioning tests [4, 21–23, 30]. A considerably larger number of studies have addressed the effectiveness of LDN and HBN. According to a study by Koo et al. on 54 patients with HSC-BPPV, directional LDN was observed in 75% of geotropic and 80% of apogeotropic types [22]. In their retrospective study, the design of which was similar to that of our study, lateralization effectiveness of LDN appeared to be superior to that in our study. In a prospective study by Han et al. on 152 patients, directional LDN occurred in 56 (96.6%) out of 58 patients with LDN [21]. In Lee et al.’s measurements (using video-oculography) of DCHN during HRT, LDN and HBN in 54 HSC-BPPV patients, lateralization rates were 82.9% (geotropic, 73.7%; apogeotropic, 93.8%) and 87.8% (geotropic, 88.9%; apogeotropic, 93.8%) in LDN and HBN, respectively, among 45 patients showing asymmetric DHCN during HRT [4].

In our study, lateralization rates of LDN and HBN were 55.9% (geotropic, 63.2%; apogeotropic, 46.7%) and 73.7% (geotropic, 73.9%; apogeotropic, 73.3%), respectively. Moreover, lateralization rates of LDN and HBN were 33.3% (geotropic, 33.3%; apogeotropic, 33.3%) and 30.0% (geotropic, 20.0%; apogeotropic, 40.0%), respectively, in patients with high probability of lateralization based on caloric tests, previous history of vestibular disease, and conversion to other semicircular canal BPPVs; these results differed from the results of previous studies [4, 21, 22]. Among 50 patients, LDN and HBN were not elicited in 16 (32%) and 12 (24%) patients. According to Koo et al., LDN was not seen in 16 (57%) out of 28 patients with the geotropic type, and in 6 (23%) out of 26 patients with the apogeotropic type [22]. In a study by Han et al., LDN was found in only 58 out of 152 patients with HSC-BPPV [21]. Moreover, LDN and HBN were not seen in 10 and 12 patients, respectively, among 45 patients in a study by Lee et al. performed using video-oculography [4]. Therefore, whether LDN and HBN are useful for lateralization in HSC-BPPV remains unclear.

**Figure 3:**
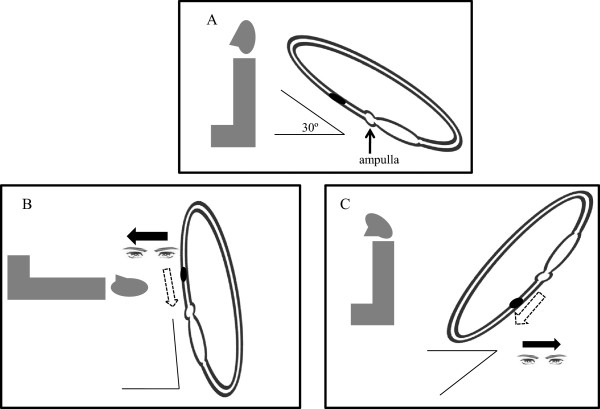
**Apogeotropic canaloithiasis in the short arm of the right horizontal semicircular canal.** It can cause ampullopetal endolymphatic migration, which induces ipsilesional (opposite directional) lying-down nystagmus **(A, B)**. It can also lead to the contralesional (opposite directional) head-bending nystagmus **(A, C)**. (Adapted from reference [23]: G. Asprella-Libonati. Pseudo-Spontaneous Nystagmus: a new sign to diagnose the affected side in Lateral Semicircular Canal Benign Paroxysmal Positional Vertigo. Acta otorhinolaryngologica Italica : organo ufficiale della Societa italiana di otorinolaringologia e chirurgia cervico-facciale, Figure one, three 2008, 28(2):73–78., with permission, Copyright © by Società Italiana di Otorinolaringologia e Chirurgia Cervico-Facciale Via Luigi Pigorini, 6/3 00162 Roma, Italy).

According to the findings of previous studies, apogeotropic nystagmus is generally induced by cupulolithiasis, but this could be manifested by particles in the short arms of semicircular canals [30, 31]. Furthermore, canalolithiasis may be converted to cupulolithiasis during HBN induction [4, 30]. These findings suggest that opposite directional LDN and HBN could be induced if dense canalith particles adhere to the cupula (Figure 3), and LDN and HBN may not be detectable with nystagmus offset resulting from canalith particles evenly distributed in the short arm and the long arm of horizontal semicircular canal. In our study, the lateralization rates of LDN and HBN were lower in apogeotropic than in geotropic nystagmus, in line with the above findings (Table 1). Moreover, LDN may not be evoked due to long arm canalolithiasis in the gravity-dependent position, since the plane of the horizontal semicircular canal is 30° off the horizontal during normal upright posture. We examined the history of vestibular neuritis and Meniere’s disease based on EMR, and no history was found in any patients. However, 13 patients had ipsilesional caloric hypofunction in both sets of the caloric test. Of the 13 patients, directional LDN and HBN were found in 53.8%, showing an insignificant difference with all patients. Therefore, LDN and HBN may be undetectable when nystagmus is offset by existing vestibulopathy. In addition, those may have be not seen since otoliths have been repositioned spontaneously during a positioning test.

This study evaluated nystagmus in all patients with HSC-BPPV using Frenzel goggles, which are conveniently used at bedside, and the outcomes were compared to those of video-oculography. Moreover, this study was able to assess the effectiveness of LDN and HBN in lateralization of the lesion side after clarifying lateralization using laboratory and clinical findings, and previous history of vestibulopathy. We found that LDN and HBN did not seem to predict lateralization of the lesion side in patients with HSC-BPPV clinically. LDN and HBN were not elicited in a large number of patients. The frequency of lateralization to the wrong direction was high. Since this study was performed retrospectively, therapeutic methods and their efficacy cannot be thoroughly examined. According to Choung et al., DCHN detected in HRT as well as Bow and Lean tests (BLT) indicated different lesion sides in patients with HSC-BPPV. When a repositioning maneuver was performed after lateralization of the affected side based on BLT, symptoms were improved [32]. Moreover, a repositioning maneuver depending on LDN and HBN also improved symptoms in 7 out of 9 patients with a symmetric DHCN on HRT in a study by Lee et al. [4]. Therefore, the analysis of treatment methods and its efficacy is crucial in evaluation of lateralization efficacy. In addition, there were only 14 patients with high probability of lateralization. The small sample size could have limited the evaluation of the lateralization rates of LDN and HBN.

**Figure 4:**
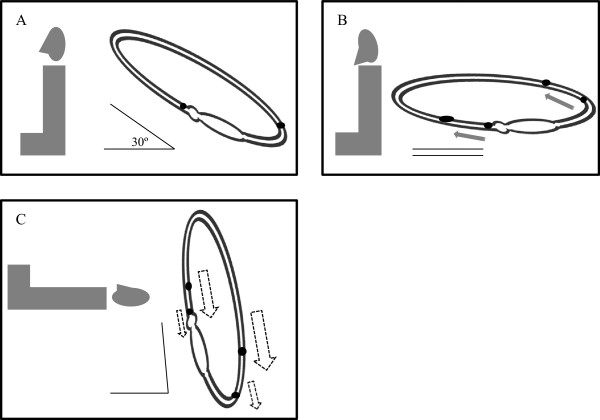
**Thirty-degree head flexion from the pitch plane.** When it is performed before the lying-down test **(A, B)** in right horizontal semicircular canal benign paroxysmal positional vertigo (HSC-BPPV), more intense lying-down nystagmus is elicited due to greater angular acceleration **(C)**. (Adapted from reference [23]: G. Asprella-Libonati. Pseudo-Spontaneous Nystagmus: a new sign to diagnose the affected side in Lateral Semicircular Canal Benign Paroxysmal Positional Vertigo. Acta otorhinolaryngologica Italica : organo ufficiale della Societa italiana di otorinolaringologia e chirurgia cervico-facciale, Figure one, three 2008, 28(2):73–78., with permission, Copyright © by Società Italiana di Otorinolaringologia e Chirurgia Cervico-Facciale Via Luigi Pigorini, 6/3 00162 Roma, Italy).

The directions of LDN and HBN are still commonly used in lateralization in patients with HSC-BPPV, especially in case of symmetric DCHN during HRT. To increase the lateralization rate of LDN and HBN, conventional maneuvers for LDN and HBN induction need to be modified. Since the horizontal semicircular canal is tilted up the horizontal plane, cupulolith and canalith particles move to the short arm of the horizontal semicircular canal upon bending the head forward. Consequently, angular acceleration increases and more intense LDN can be elicited due to greater angular acceleration (Figure 4). In addition, direction-changing nystagmus is reported to be intense after head shaking [30], thus this methods can be taken into consideration.

## Conclusions

Lying-down nystagmus and head-bending nystagmus do not seem to predict lateralization in patients with horizontal semicircular canal benign paroxysmal positional vertigo. To improve the lateralization rate in HSC-BPPV, maneuvers for eliciting LDN or HBN need to be modified and prospective studies using other lateralizing tests are crucial in the future.
